# MARCKS N-terminal sequence-derived inhibitor peptides impair monocytic ROS production and migration *via* MARCKS-independent effects

**DOI:** 10.1007/s11033-025-11027-8

**Published:** 2025-09-17

**Authors:** Friederike Kühl, Jana Lea Kopper, Lena Sofie Wriede, Bastian Welz, Ralf Lichtinghagen, Korbinian Brand, René Huber

**Affiliations:** Hannover Medical School, Institute of Clinical Chemistry and Central Laboratory, Carl-Neuberg-Str. 1, 30625 Hannover, Germany

**Keywords:** MARCKS, Monocytes, Knock-out, MANS, BIO-11006, ROS, Migration

## Abstract

**Background:**

Myristoylated alanine-rich C kinase substrate (MARCKS) is a versatile unstructured protein involved in numerous cellular processes and associated with various diseases. In this study, the effect of MARCKS’ N-terminal sequence-derived inhibitor peptides MANS (“myristoylated N-terminal sequence”) and BIO-11006 on monocytic ROS production and migration was assessed.

**Methods and results:**

Stimulation of calcitriol-differentiated monocytic THP-1 cells with PMA, opsonized (ops.) *E. coli*, ops. *S. aureus*, and ops. zymosan led to considerable ROS production (as determined using a chemiluminescence-based assay), an effect significantly reduced in THP-1-derived MARCKS knock-out (KO) cells that were generated with the CRISPR/Cas9 technique. MANS similarly inhibited ROS production in monocytic THP-1 and PLB-985 cells as well as primary human monocytes induced by various stimuli, while BIO-11006 predominantly affected PMA-induced ROS levels. TNF preincubation enhanced monocytic ROS production, but was not able to compensate for MANS treatment or MARCKS deficiency. Unexpectedly, an inhibition of ROS formation by both inhibitor peptides could also be observed in MARCKS KO cells, indicating a target-independent effect of MANS and BIO-11006 at least in MARCKS-deficient monocytic cells. Comparable negative effects of MANS in both WT and KO cells could also be observed when monocytic migration was assessed in transwell assays.

**Conclusion:**

Our data suggest that MARCKS inhibitor peptides MANS and (to a lesser extent) BIO-11006 are able to inhibit MARCKS-associated cellular processes in monocytic cells by MARCKS-independent mechanisms.

## Introduction

The ubiquitously expressed protein MARCKS (myristoylated alanine-rich C kinase substrate) is an acidic protein comprising 332 amino acids (aa). Despite being a predominantly unstructured molecule, it includes a myristoylated N-terminal domain, a MARCKS homology 2 (MH2) domain, and a lysine-rich effector domain (ED) possessing a phosphorylation site with four serine residues. The myristoyl group and the ED are responsible for the association of unphosphorylated MARCKS with cellular membranes, while ED phosphorylation (e.g., by protein kinase C) leads to its translocation to the cytosol. The latter can also be achieved *via* binding of calmodulin [1, 2].

In dependency of its localization, MARCKS is involved in versatile effects. At the membrane, for instance, MARCKS mediates actin crosslinking [3], sequesters phosphatidylinositol 4,5-bisphosphate (PIP_2_) [4], and binds to vesicle traffic regulating proteins [5], while its dissociation renders PIP_2_ available for the activation of downstream signaling including the phosphatidylinositol 3-kinase (PI3K)/Akt pathway [6]. Thus, MARCKS contributes to the regulation of central cellular processes involving rearrangements of the cytoskeleton (e.g., mitosis, adhesion, migration), intracellular transportation (e.g., vesicle trafficking, degranulation, or secretion), and signal transduction/gene expression [7–9]. Moreover, we have recently shown in the monocytic cell type that MARCKS is an essential regulator of total as well as intracellular reactive oxygen species (ROS) production [10], an effect that may involve the PI3K/Akt cascade [11], and prone to increased phosphorylation in the linker region (connecting MH2 and ED) following long-term TNF treatment [12]. Accordingly, disturbances in MARCKS level or function are associated with numerous pathophysiological conditions [13], e.g., in pulmonary diseases [14], fibrosis [15], or cancer [16].

Since MARCKS does not exhibit enzymatic activity, it cannot be inhibited using common catalytic domain-targeting small molecule inhibitors. However, it has been shown that MARCKS-derived peptides reflecting either the myristoylated N-terminal part (“myristoylated N-terminal sequence”, MANS; comprising amino acids (aa) 1–24 [14]) or the ED [17] (aa 151–175 [18]) are potent modulators of its function [19]. MANS, for instance, was characterised as a peptide inhibitor of MARCKS [20], which may enter cells *via* its hydrophobic myristoyl residue [21], though myristoylation is not crucial for its inhibitory functions [22]. MANS was applied in vitro and in vivo [23] and has been shown to suppress mucus hypersecretion in a murine model of asthma [14], to inhibit human leukocyte degranulation [24], and to decrease migration/invasion of human lung cancer cells [23]. Thus, MANS is regarded as a useful tool in the analysis of MARCKS’ functions [25]. However, due to its size, insufficient solubility, and propensity to rapid hydrolysis, MANS cannot be converted into a suitable drug [20]. Therefore, the shorter, highly soluble, and non-myristoylated analog BIO-11006 has been developed [26] comprising the active region of MANS, i.e., aa 1–10 [27]. Due to its improved solubility (achieved by replacing the myristoylation by an acetyl group) and increased resistance against hydrolysis [20], it can be applied as an aerosol, which makes it a promising candidate for the treatment of pulmonary diseases [28]. In the mouse model, positive effects of BIO-11006 on lipopolysaccharide (LPS-)induced lung injury [27], ozone-induced airway neutrophilia and inflammation [26], or methacholine-induced airway obstruction and mucus hypersecretion [28] have been described. Moreover, following successful phase I studies [20], aerosolised BIO-11006 has been tested in phase II clinical trials in patients with chronic obstructive pulmonary disease (COPD; NCT00648245), acute respiratory distress syndrome (ARDS; NCT03202394) [29], and showed promising results in patients suffering from late stage non-small cell lung cancer [30].

In earlier experiments, we demonstrated that MARCKS deficiency massively impairs ROS formation by calcitriol-differentiated monocytic THP-1-derived MARCKS knock-out (KO) cells [10]. We therefore applied MANS and BIO-11006 to monocytic cells to assess whether these peptides suppress monocytic ROS production to a similar extent as the KO. As a control, the myristoylated “random N-terminal sequence” (RNS) was used, which consists of a randomised arrangement of the aa included in MANS [21] and shows no effect on MARCKS-dependent cell functions [20]. In comparison to RNS-treated samples, MANS and BIO-11006 inhibited ROS generation in differentiated THP-1 and PLB-985 wild-type (WT) cells as well as primary human monocytes. Surprisingly, comparable effects on ROS levels could be observed in MARCKS KO cells, indicating that the inhibitor peptides used have MARCKS-independent effects. Since MANS is attributed to attenuate migration [22, 23, 31], we analysed monocyte migration in the presence of MANS in MARCKS WT and KO cells. Again, equivalent suppressive effects were measured in both WT and KO cells. Collectively, our data suggest that MARCKS inhibitor peptides MANS and BIO-11006 (also) possess target-independent activities and may inhibit MARCKS-associated cell functions by MARCKS-independent mechanisms, at least in the monocytic cell type and in the absence of MARCKS.

## Materials and methods

### CRISPR/Cas9-mediated generation of MARCKS WT and KO cells

**Fig. 1:**
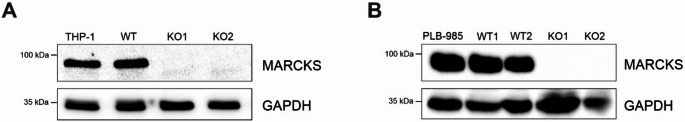
MARCKS levels in monocytic THP-1- and PLB-985-derived MARCKS WT and KO cells. THP 1- (**A**) and PLB-985-derived MARCKS KO cells (**B**) were generated using the CRISPR/Cas9 technique and MARCKS protein levels were detected in monocytic (i.e., 5 d 100 nM calcitriol-differentiated) MARCKS WT (THP-1: 1 clone, PLB-985: 2 clones) and KO cells (2 clones each). For comparison, MARCKS levels in genuine monocytic THP-1 and PLB-985 cells (i.e., without CRISPR/Cas9 treatment) are shown (Western Blot, whole cell extracts, loading control: GAPDH; representative experiments, *n* = 3)

The generation of THP-1- and PLB-985-derived MARCKS WT and KO cells using the CRISPR/Cas9 technique was performed as previously described [10]. In short, MARCKS-targeting CRISPR/Cas9 plasmids (PX458-MARCKS) were generated based on the plasmid pSpCas9(BB)−2A-GFP (PX458; plasmid #48138, Addgene, Watertown, USA) originally produced by Feng Zhang and processed according to [32] using target sequences from the Brunello library (#1: 5’-AAGAAGTCTTTCAAGCTGAG-3’, #2: 5’-CTCACCTTTCTCGGCCGCGG-3’, and #3: 5’-TCGTCGCCTTCCAAAGCGAA-3’) [33]. THP-1 or PLB-985 cells were transfected with a Nucleofector II (program T-020) using the Nucleofector Kit T (Lonza, Basel, Switzerland) according to the manufacturer’s instructions. Single clones were established by flow cytometry-based cell sorting of green fluorescent protein (GFP-)positive cells at the Hannover Medical School core facility cell sorting, resulting in the establishment of two THP-1- and two PLB-985-derived MARCKS KO clones. In the respective clones, MARCKS deficiency was confirmed by Western Blot (Fig. 1), flow cytometry, and cycle sequencing (see below). One THP-1-derived clone and two PLB-985-derived clones, in which the MARCKS alleles remained unaffected, were used as MARCKS WT controls.

### Western blot

MARCKS levels in THP-1- and PLB-985-derived WT and KO cells were determined in whole cell extracts using the Western Blot technique (Fig. 1) as described earlier [34]. For protein detection, primary antibodies targeting human MARCKS (D88D11 XP^®^; Cell Signaling, Danvers, USA) and glyceraldehyde-3-phosphate dehydrogenase (GAPDH; Sigma Aldrich, St. Louis, USA) were used. The horseradish peroxidase (HRP-)coupled secondary antibody was purchased from Cell Signaling. Visualization was performed using appropriate detection reagents (enhanced chemiluminescence (ECL), Thermo Fisher, Bonn, Germany; WesternBright Sirius, Advansta, Menlo Park, USA) and the Bio-imager ECL Chemostar (Intas, Göttingen, Germany).

### Flow cytometry

Detection of MARCKS levels by intracellular staining was performed as previously described using the MARCKS antibody D88D11 XP^®^ (Cell Signaling) and the Alexa Fluor 647-labelled AffiniPure F(ab’)₂ Fragment (Jackson ImmunoResearch, West Grove, USA). For detection, a FACSCanto II flow cytometer and the FACSDiVa software from BD Biosciences (Heidelberg, Germany) were applied. Data were analysed and illustrated with FlowJo (BD Bioscience) and GraphPad PRISM 5.02 (GraphPad Software, La Jolla, USA) [10].

### Cycle sequencing

Integrity of MARCKS alleles following the CRISPR/Cas9 procedure was assessed using cycle sequencing as described in [10]. Genomic DNA from MARCKS WT and KO cells was isolated using the QIAamp DNA Mini Kit (Qiagen, Hilden, Germany). Concentration and purity were confirmed with the Nanodrop ND-1000 (PeqLab, Erlangen, Germany). Targeted DNA sequences were amplified using the primers 5’-AGCTGCAGGCCAACGGCAGCGC-3’ and 5’-TGCGCCCCCGGCGGCCTCGT-3’ (for MARCKS KO #1 and #2) or 5’-TGTTTCCCCTCTTGGATCTGT-3’ and 5’-TCCACGAATGAGCCTTGGGA-3’ (for MARCKS KO #3) and cloned into the vector pCR-Blunt II-TOPO using the Zero Blunt TOPO PCR cloning kit (Invitrogen, Darmstadt, Germany). Cycle sequencing was performed at Eurofins Genomics (Ebersberg, Germany). Sequencing data were analysed and processed using Chromas (Technelysium, South Brisbane, Australia), CLC Sequence Viewer 8 (Qiagen), and SnapGene Viewer 4.1.1 (GSL Biotech, Chicago, USA).

### Isolation of primary monocytes

Whole Blood samples were collected from healthy donors in lithium heparin collection tubes following informed consent at the Institute of Clinical Chemistry and Central Laboratory, Hannover Medical School. The experiments were approved by the Hannover Medical School ethics committee (9783_BO_K_2021) and conducted in accordance with the Declaration of Helsinki. First, peripheral blood mononuclear cells (PBMCs) were isolated from whole blood by density gradient centrifugation (1,000 x g, 20 min) in Leucosep™ tubes (Greiner, Frickenhausen, Germany) using BioColl^®^ separation solution (Bio&Sell, Nürnberg, Germany). Subsequently, monocytes were isolated with the Pan Monocyte Isolation Kit (Miltenyi, Bergisch Gladbach, Germany) according to the manufacturer’s instructions [35]. Purity of the primary human monocytes was confirmed with the XN-10TM Hematology Analyzer (Sysmex, Norderstedt, Germany) using the body fluid measurement program.

### Cell culture

Human THP-1 and PLB-985 cells were purchased from the Deutsche Sammlung von Mikroorganismen und Zellkulturen (DSMZ, Braunschweig, Germany). Primary monocytes, THP-1, PLB-985, and THP-1- as well as PLB-985-derived MARCKS WT and KO cells were cultured in Roswell Park Memorial Institute (RPMI)1640 medium including 100 U/ml penicillin, 100 mg/ml streptomycin (Biochrom, Berlin, Germany), and 7.5% fetal calf serum (FCS; Sigma Aldrich). Cultures of primary monocytes were additionally supplemented with 2% oxal-acetate/pyruvate/insulin (OPI) media supplement (Sigma Aldrich) and 1% minimum essential medium non-essential amino acids solution (Thermo Fisher) [35]. For experiments involving long-term TNF preincubation, THP-1 cells were incubated for 48 h with 80 ng/ml TNF (Peprotech, Rocky Hill, USA) as described before [36].

For fundamental objectives, we performed a higher number of independent experiments (up to *n* = 11) to comprehensively demonstrate the basic effects. For additional/supportive analyses, *n* = 3–5 independent experiments were performed.

### Cell differentiation

Differentiation of THP-1, PLB-985, and THP-1-/PLB-985-derived MARCKS WT and KO cells towards a monocytic phenotype was performed for 5 days with 100 nM calcitriol (Peprotech) [37]. Differentiation of PLB-985 as well as PLB-985-derived MARCKS WT and KO cells towards a neutrophilic phenotype was performed for 3 days with 1.25% DMSO (Sigma Aldrich) [38]. Differentiation was confirmed by flow cytometry (Alexa Fluor 647-coupled CD14 and phycoerythrin-coupled CD11b antibodies; Biolegend, San Diego, USA).

### Inhibitor and control peptides

MANS (myristic acid–GAQFSKTAAKGEAAAERPGEAAVA), RNS (myristic acid–GTAPAAEGAGAEVKRASAEAKQAF) [21], and BIO-11006 (acetyl-GAQFSKTAAK) [26] were purchased from MedChemExpress (Sollentuna, Sweden).

### ROS detection

ROS formation was induced by 100 nM phorbol 12-myristate 13-acetate (PMA; Sigma Aldrich), opsonised (ops.) TOP10 *E. coli* (Invitrogen), ops. *S. aureus* (Invivogen, Toulouse, France), or ops. zymosan (Sigma Aldrich). For opsonization, methanol-fixed *E. coli*, heat-killed *S. aureus,* or zymosan were washed with Hank’s balanced salt solution (HBSS) with magnesium and calcium (Thermo Fisher), centrifuged (8,000 x g, 2 min), and incubated for 30 min (shaking, 600 rpm) with pooled human complement serum (Innovative Research, Peary Court, USA). Afterwards, ops. particles were washed and diluted in HBSS. For subsequent ROS assays, final concentrations of at least five bacteria per cell or 150 µg/ml zymosan were prepared.

Total ROS were detected using an Orion L microplate luminometer (Berthold, Pforzheim, Germany) by ROS-dependent development of chemiluminescence, i.e., the conversion of luminol (5-amino-2,3-dihydro-1,4-phtalazinedione) in the presence of ROS [10]. 2 × 10^5^ THP-1-derived cells or 3 × 10^5^ PLB-985-derived cells (WT vs. KO) were seeded in HBSS (50 µl; 30 min, 37 °C) in white Costar 96 well plates (Corning, Corning, USA). Depending on the experimental setup, MARCKS-inhibiting peptides were added (100 µM MANS or RNS or 10 − 1,000 µM BIO-11006) and cells were preincubated for 30 min at 37 °C, 5% CO_2_. Subsequently, 50 µl of 2 x ROS induction/detection solution (0.1 mM luminol, 1 U/ml horseradish peroxidase (for THP-1 cells only; both Sigma Aldrich), and the respective ROS-inducing stimulus in HBSS) were added. Analysis and visualization of ROS data were performed using GraphPad PRISM 5.02. Data were presented as relative light units (RLU) over time from which the area under the curve (AUC) was generated describing the total amount of ROS generated during the experiment.

### Transwell migration assay

Chemotaxis was measured using Transwell^®^ 96-well plates with 5 μm pore polycarbonate membranes (Corning). Therefore, calcitriol-differentiated THP-1 (2 × 10^5^) or PLB-985 cells (3 × 10^5^) were seeded in 100 µl pure RPMI per transwell. Depending on the experiment, peptide inhibitors were directly added to the cells in the upper transwell chamber followed by preincubation for 30 min at 37 °C. Subsequently, RPMI ± chemoattractant was added to the lower chamber of the transwell and cells were incubated for 4 h at 37 °C, 5% CO_2_. MCP-1 (50 ng/ml; Peprotech), LTB_4_ (100 ng/ml; Cayman Chemicals, Ann Arbour, USA), and FCS (10%; PAN-Biotech, Aidenbach, Germany) served as chemoattractants. Relative numbers of transmigrated cells were determined with an ATP assay as follows: the cell suspensions in the lower transwell chambers were transferred to a white, opaque 96-well plate (Corning); cells attached to the lower side of the transwell membrane were detached using accutase (Biolegend) and transferred to the same wells. Plates were centrifuged (400 x g, 5 min) and supernatant was carefully removed until a final volume of 100 µl per well was achieved. Then, CellTiter-Glo^®^ 2.0 Cell Viability Assay reagent (100 µl; Promega, Mannheim, Germany) was added, the cells were resuspended (shaking, 450 rpm, 2 min, protected from light), and finally incubated at room temperature for further 10 min. Luminescence signals were measured for 1 s per well in the Orion L luminometer.

### Statistical analysis

Data were depicted as means ± standard deviation (SD). For ROS experiments, Gaussian distribution of the values was demonstrated using the Shapiro-Wilk test and the paired two-tailed ratio t test or the unpaired t test with Welch’s correction was applied for determining statistically significant differences among different groups (GraphPad Prism 5.02).

## Results

### ROS production is inhibited in monocytic MARCKS KO cells

**Fig. 2:**
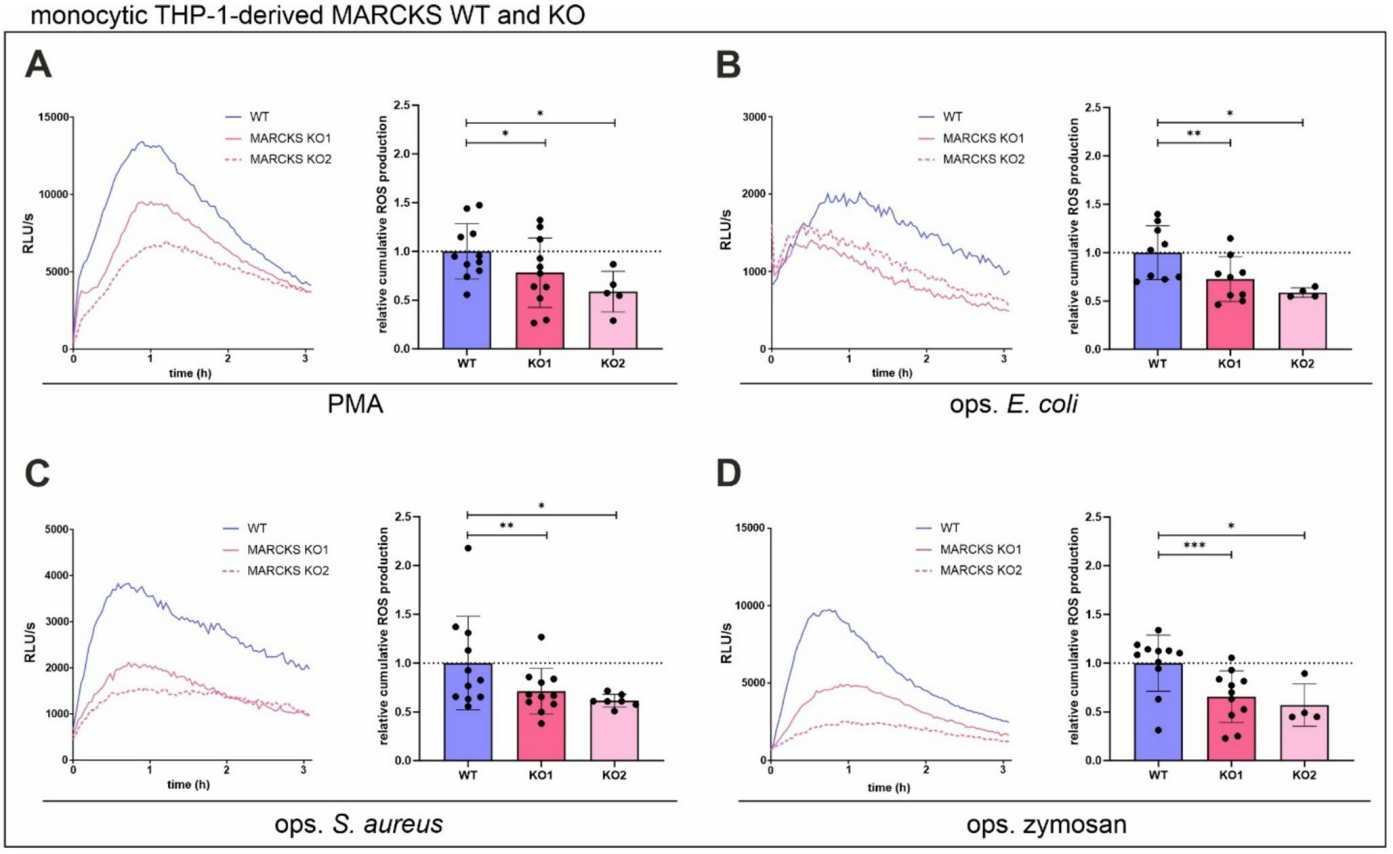
ROS production is significantly inhibited in monocytic MARCKS KO cells. (**A–D**) PMA-, *E. coli-*,* S. aureus*-, and zymosan-induced ROS production is inhibited in monocytic THP-1-derived MARCKS KO cells. Kinetics of total (i.e., intra- and extracellular) ROS generation (representative experiments) and cumulative total ROS production (i.e., the area under the curve (AUC) within 3 h; mean ± SD, *n* = 4–11) by monocytic THP-1-derived MARCKS WT and KO clones (2 × 10^5^ cells) in response to 100 nM PMA (**A**), opsonised (ops.) *E. coli* (**B**), ops. *S. aureus* (**C**; at least 5 bacteria per cell), and ops. zymosan (**D**; 150 µg/ml). Mean cumulative total ROS levels in WT cells was set as 1 (dotted line). KO1 and KO2 represent distinct MARCKS KO clones. *p ≤ 0.05; **p ≤ 0.01; ***p ≤ 0.005; paired two-tailed ratio t test

Since we have demonstrated before that MARCKS deficiency similarly reduces both total and intracellular ROS production by monocytic THP-1 cells in response to PMA and ops. (gram-negative) *E. coli* [10], we wanted to figure out whether equivalent effects can be observed in the presence of further ROS-inducing stimuli. Therefore, calcitriol-differentiated THP-1-derived MARCKS WT and KO cells were stimulated with PMA and ops. *E. coli* (for comparison), ops. (gram-positive) *S. aureus* bacteria, and ops. zymosan. First, the significant reduction of PMA- (Fig. 2A) and *E. coli*-induced total ROS formation (Fig. 2B) in monocytic MARCKS KO cells described before was reproduced. Following stimulation with both ops. *S. aureus* (Fig. 2C) and zymosan (Fig. 2D), a similar reduction of ROS levels was observed in MARCKS-deficient cells when compared to the WT, thus confirming that the absence of MARCKS considerably impairs monocytic ROS production induced by various stimuli.

### ROS production is inhibited by MARCKS inhibitor peptide MANS

**Fig. 3:**
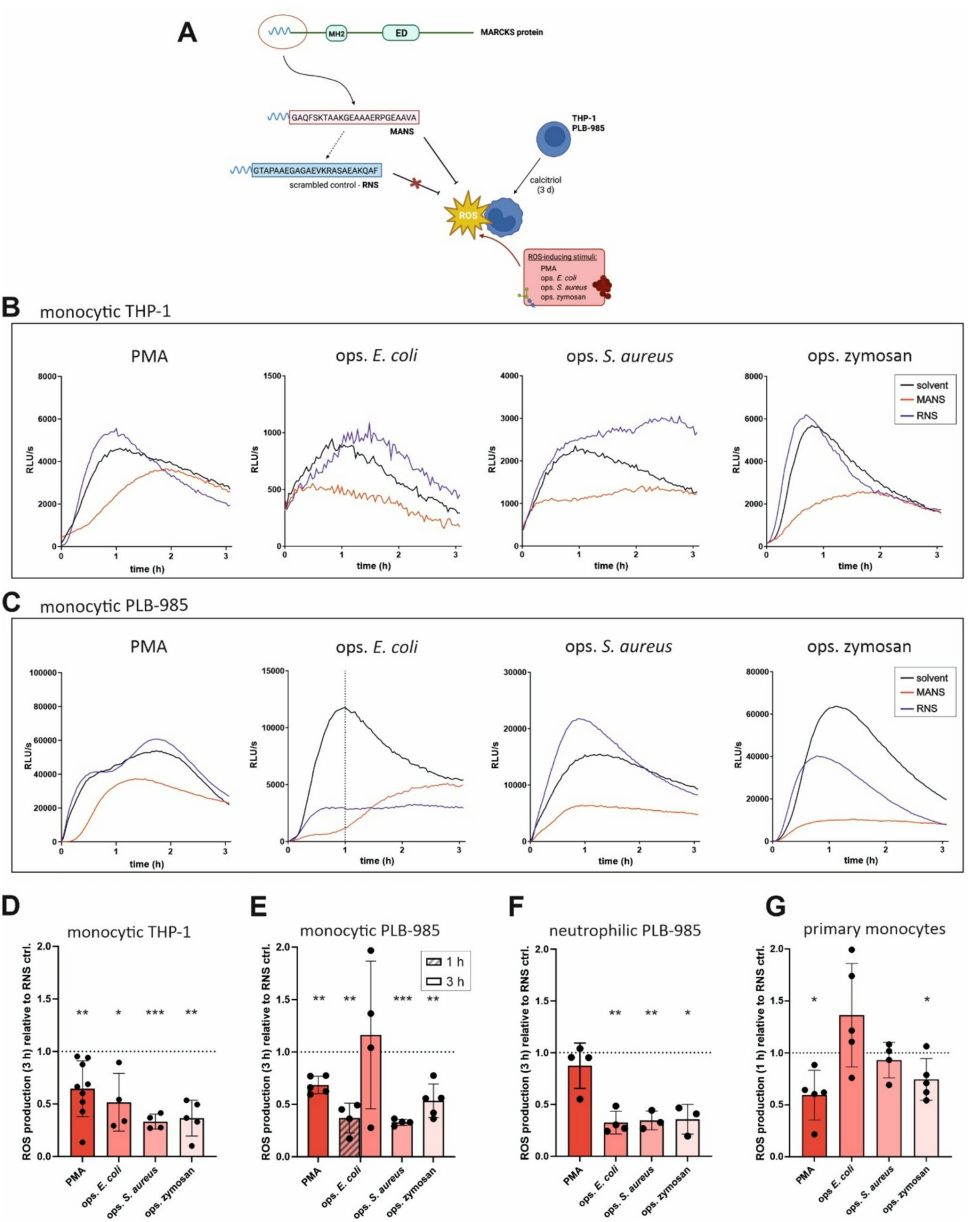
MANS inhibits monocytic and neutrophilic ROS production. (**A**) Experimental design. Differentiated THP-1 (2 × 10^5^) and PLB-985 cells (3 × 10^5^) were preincubated with 100 µM MANS peptide or the scrambled control peptide RNS. ROS production was induced by 100 nM PMA, ops. *E. coli*, ops. *S. aureus* (at least 5 bacteria per cell), or 150 µg/ml ops. zymosan, and the induced ROS generation was measured for 3 h. Created with BioRender.com. (**B**) MANS inhibits ROS production in monocytic THP-1 cells. Kinetics of total ROS generation (representative experiments) of monocytic THP-1 cells in response to PMA, ops. *E. coli* and *S. aureus* bacteria, or ops. zymosan following pretreatment with MANS or RNS. (**C**) MANS inhibits ROS production in monocytic PLB-985 cells. Kinetics of total ROS generation (representative experiments) of MANS- or RNS-pretreated monocytic PLB-985 cells in response to PMA or ops. *E. coli*, *S. aureus*, and zymosan. (**D**, **E**) Cumulative total ROS production (mean ± SD) of the experiments performed as described in B (*n* = 3–8) and C (*n* = 3–4), respectively. (**F**, **G**) MANS inhibits ROS production in neutrophilic PLB-985 cells and primary human monocytes. Cumulative total ROS production of MANS- or RNS-pretreated neutrophilic (i.e., 3 days 1.25% DMSO-differentiated) PLB-985 cells (**F**) and primary human monocytes (2 × 10^5^, ROS measurement: 1 h; **G**) in response to the indicated stimuli (mean ± SD, *n* = 3–5). Cumulative total ROS levels in the respective RNS-treated controls were set as 1 (dotted line). * *p* ≤ 0.05; ** *p* ≤ 0.01, *** *p* ≤ 0.005 in comparison to the RNS control; unpaired t test with Welch’s correction

As a next step, the effect of the MARCKS inhibitor peptide MANS on total ROS formation was analysed. Calcitriol-differentiated THP-1 and PLB-985 cells were preincubated with MANS or the scrambled control peptide RNS, subsequently stimulated with PMA, ops. *E. coli*, ops. *S. aureus*, or ops. zymosan, and the resulting ROS generation was measured (Fig. 3A). Monocytic THP-1 (Fig. 3B, D) and PLB-985 cells (Fig. 3C, E) exhibited significantly reduced ROS production in the presence of MANS when compared to RNS, irrespective of the ROS-inducing stimulus used. In ops. *E. coli*-stimulated monocytic PLB-985 cells, however, the significant reduction was limited to the first hour of stimulation (dotted line in Fig. 3C) and levelled out afterwards due to high variation at later time points and an inhibitory effect of RNS in this set of experiments (Fig. 3E). In PLB-985 cells that were differentiated with DMSO towards the neutrophilic cell type, an equivalent MANS-dependent decrease could be observed for ops. *E. coli*-, *S. aureus-*, or zymosan-provoked ROS generation (Fig. 3F). Moreover, ROS generated by primary human monocytes in response to PMA and ops. zymosan were significantly reduced by MANS (Fig. 3G). These data indicate that the application of the MARCKS inhibitor peptide MANS is able to mimic the ROS suppressing effect of MARCKS deficiency in cells of the myeloid lineage.

### ROS production is inhibited by MANS in both MARCKS WT and KO cells

**Fig. 4:**
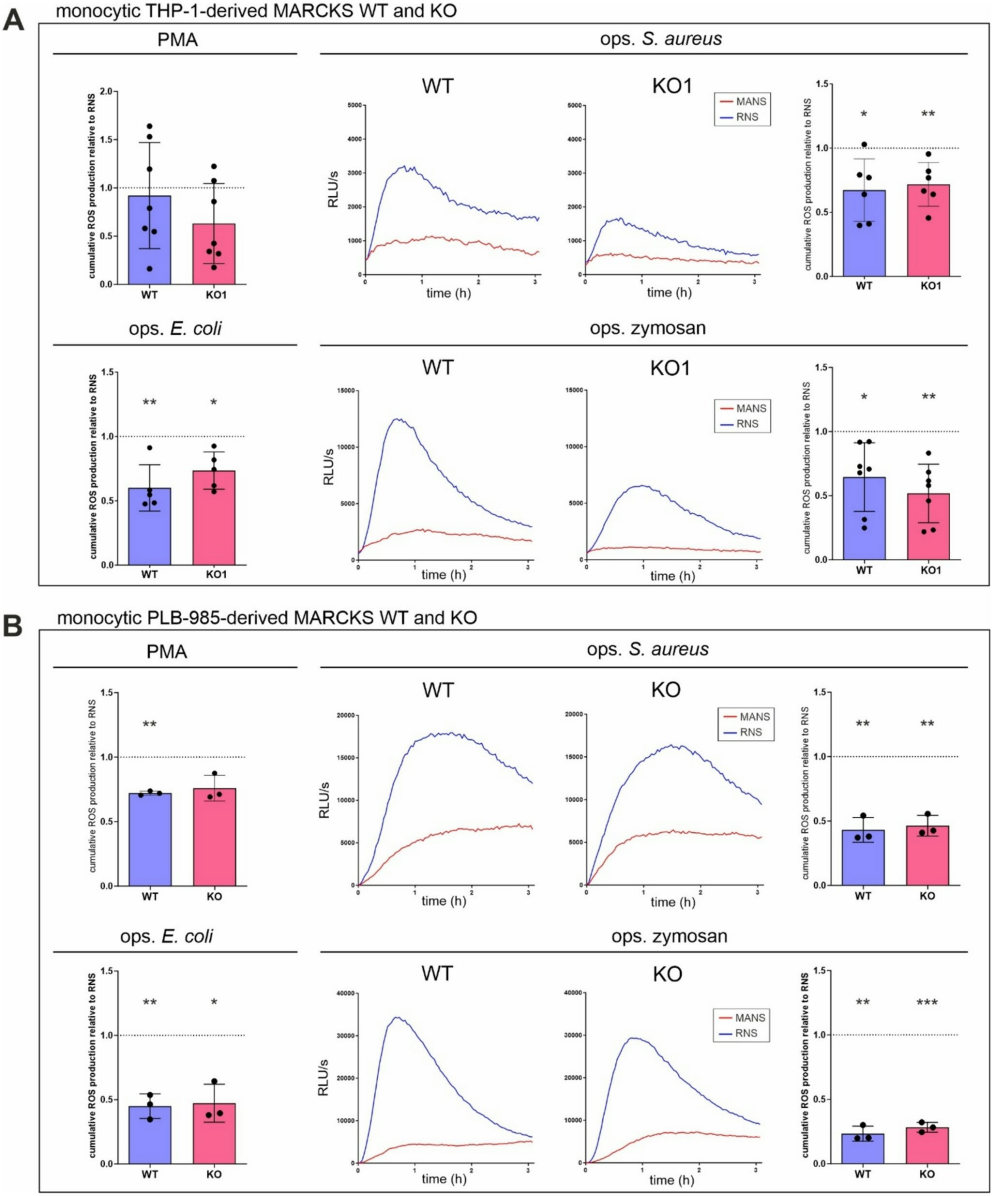
MANS inhibits ROS production in both MARCKS WT and KO cells. (**A**) MANS inhibits ROS production in monocytic THP-1-derived WT and KO cells. Cumulative total ROS production in response to 100 nM PMA (*n* = 7) and ops. *E. coli* (at least 5 bacteria per cell, *n* = 5) in 100 µM MANS-pretreated monocytic THP-1-derived MARCKS WT and KO clones (2 × 10^5^ cells; mean ± SD). Kinetics of total ROS generation (representative experiments) and cumulative total ROS levels of ops. *S. aureus*- (at least 5 bacteria per cell, *n* = 6) and ops. zymosan-stimulated (150 µg/ml, *n* = 7) MARCKS WT and KO clones following MANS pretreatment (mean ± SD). (**B**) MANS inhibits monocytic ROS production of monocytic PLB-985-derived WT and KO cells. Cumulative total ROS production in response to PMA and ops. *E. coli* in MANS-pretreated monocytic PLB-985-derived MARCKS WT and KO clones (3 × 10^5^; mean ± SD, *n* = 3, each data point represents the mean of WT1 and WT2 or KO1 and KO2, respectively). Kinetics of total ROS generation (representative experiments) and cumulative total ROS levels in MANS-pretreated and ops. *S. aureus*- and ops. zymosan-stimulated MARCKS WT and KO clones (mean ± SD, *n* = 3, each data point represents the mean of WT1 and WT2 or KO1 and KO2, respectively). Cumulative total ROS levels in the respective RNS-treated controls were set as 1 (dotted line). *p ≤ 0.05; **p ≤ 0.01 in comparison to the RNS control; unpaired t test with Welch’s correction

Initially intended as a negative control, the effect of MANS on total ROS production was assessed in THP-1- and PLB-985-derived MARCKS KO cells, with the respective WT cells as the corresponding positive control. Therefore, MARCKS WT and KO THP-1 cells were preincubated with RNS or MANS before ROS production was activated. As expected, MANS reduced PMA-, ops. *E.coli*-, ops. *S. aureus*-, and ops. zymosan-induced ROS generation (Fig. 4A) in MARCKS WT cells in comparison to RNS-treated cells. Surprisingly, the same inhibitory effect was observed when MANS was applied to the assumed negative control cells: despite the absence of its supposed target, MANS inhibited ROS generation by THP-1-derived MARCKS KO cells in response to the four stimuli (Fig. 4A). This unexpected impact of MANS on ROS in the absence of MARCKS was also observed, when PLB-985-derived MARCKS KO cells were used and stimulated with PMA, ops. *E. coli*, ops. *S. aureus*, and ops. zymosan (Fig. 4B).

### TNF preincubation enhances monocytic ROS production, but does not compensate for MANS treatment and/or MARCKS deficiency

**Fig. 5:**
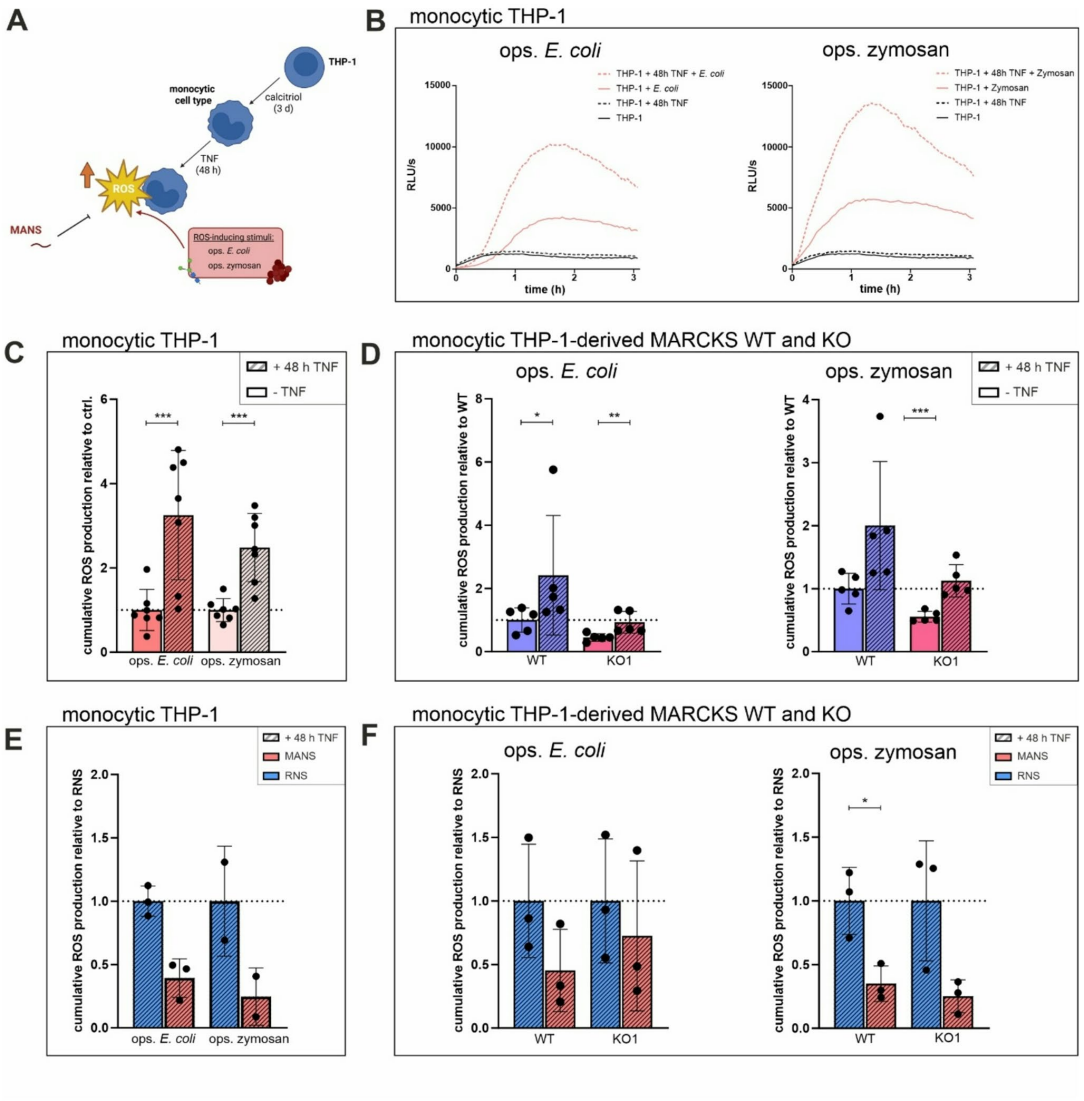
TNF preincubation enhances monocytic ROS production, but does not compensate for MANS treatment and/or MARCKS deficiency. (**A**) Experimental design. 2 × 10^5^ monocytic THP-1 or THP-1-dervied MARCKS WT and KO cells were preincubated ± 80 ng/ml TNF for 48 h and 100 µM MANS or RNS for 30 min. ROS production was induced by ops. *E. coli* (at least 5 bacteria per cell) or 150 µg/ml ops. zymosan, and the induced ROS generation was measured. Created with BioRender.com. (**B**) TNF pretreatment enhances ROS production in monocytic THP-1 cells. Kinetics of total ROS production (representative experiments) of TNF-preincubated monocytic THP-1 cells in response to ops. *E. coli* and ops. zymosan. (**C**) Cumulative total ROS production of experiments performed as described in B (mean ± SD, *n* = 7). (**D**) TNF pretreatment enhances ROS production in monocytic THP-1-derived MARCKS WT and KO cells. Cumulative total ROS production in response to ops. *E. coli* and ops. zymosan in TNF-preincubated monocytic THP-1-derived MARCKS WT and KO clones (mean ± SD, *n* = 5). Cumulative total ROS levels in TNF-free THP-1 (C) or WT controls (D) were set as 1 (dotted line). (**E**) MANS inhibits monocytic ROS production in TNF-preincubated monocytic THP-1 cells. Cumulative total ROS production of TNF-preincubated and MANS-pretreated monocytic THP-1 cells in response to ops. *E. coli* and ops. zymosan (mean ± SD, *n* = 2–3). (**F**) MANS inhibits monocytic ROS production in TNF-preincubated monocytic THP-1-derived WT and KO cells. Cumulative total ROS production of TNF-preincubated and MANS-pretreated monocytic THP-1-derived WT and KO cells in response to ops. *E. coli* and ops. zymosan (mean ± SD, *n* = 3 each). Cumulative total ROS levels in RNS-treated cells were set as 1 (dotted line). *p ≤ 0.05; **p ≤ 0.01, ***p ≤ 0.005; paired two-tailed ratio t test

In earlier experiments, we have shown that long-term TNF preincubation primes monocytic cells thus resulting in significantly increased PMA- and ops. *E. coli*-dependent ROS levels, while having no compensatory influence on reduced ROS formation in PMA- or ops. *E. coli*-stimulated MARCKS KO cells [10]. Therefore, the impact of TNF on ops. *E. coli*- and zymosan-induced ROS production under conditions of MARCKS deficiency and/or MANS treatment should be tested (Fig. 5A). Consistent with our previous data, pure TNF preincubation (i.e., without further ROS-inducing stimulation) for 48 h did not elevate basic ROS levels in monocytic THP-1 cells, but significantly enhanced ops. *E. coli*- and zymosan-dependent ROS production (Fig. 5B, C) when compared to cells without TNF preincubation. An equivalent effect was observed in TNF-preincubated THP-1-derived MARCKS WT and KO cells following ops. *E. coli* and zymosan stimulation (Fig. 5D). In TNF preincubated monocytic THP-1 cells (Fig. 5E) as well as THP-1-derived MARCKS WT and KO cells (Fig. 5F), MANS reduced ROS formation in response to both stimuli, again with no obvious differences between MANS-treated WT and KO cells. Together, these results further support the assumption that TNF primes monocytic cells to enable enhanced ROS formation, but has no compensatory effect on reduced ROS levels in the absence of MARCKS. They also indicate that TNF does not differentially modulate MANS-mediated effects in MARCKS WT and KO cells.

### PMA-induced ROS production is reduced by BIO-11006 in MARCKS WT and KO cells

**Fig. 6:**
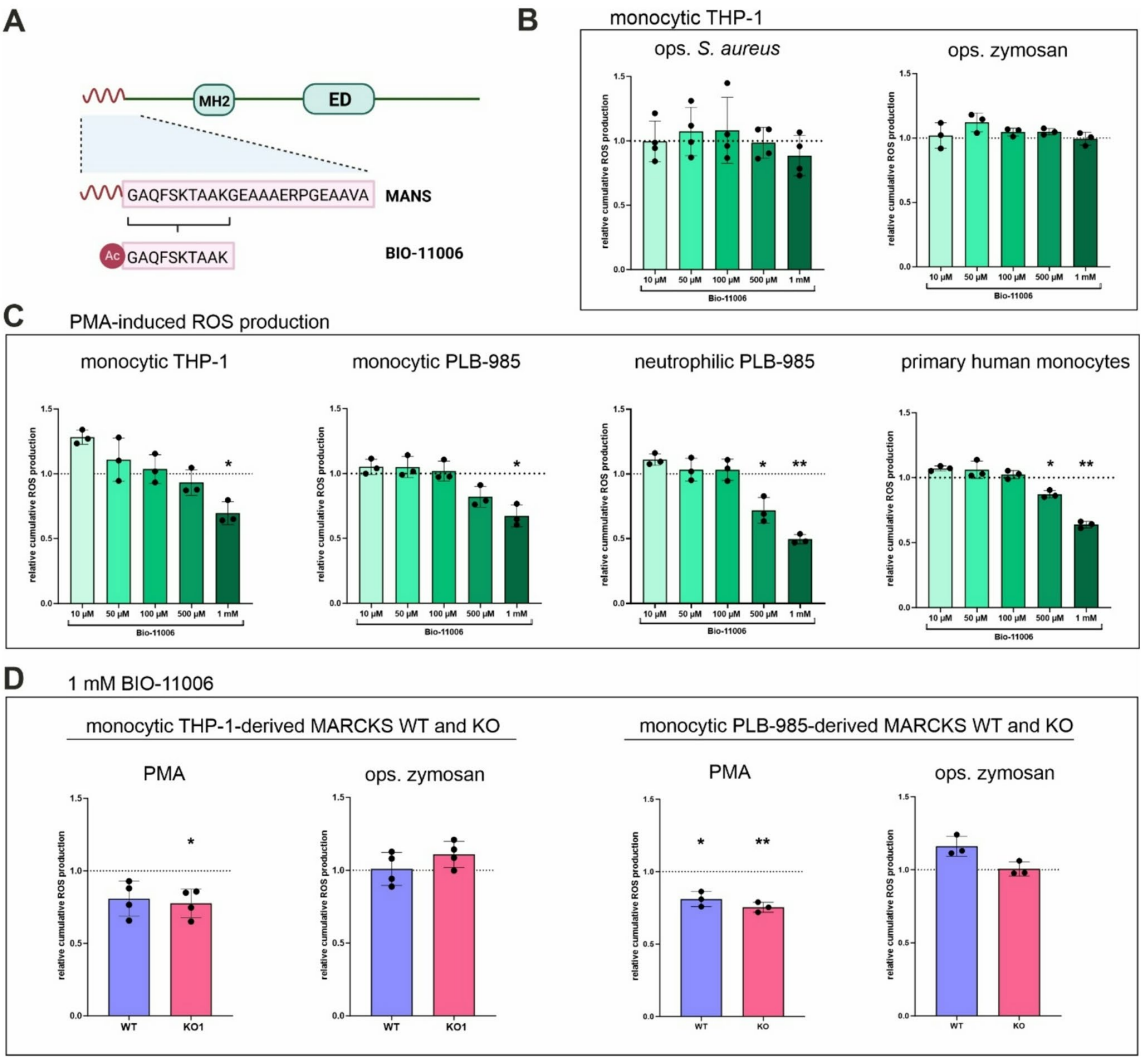
BIO-11006 inhibits PMA-induced ROS production in MARCKS WT and KO cells. (**A**) Schematic representation of the MANS-derived peptide BIO-11006 (Ac - acetylation). Created with BioRender.com. (**B**) BIO-11006 has no effect on ops. *S. aureus-* and zymosan-induced ROS production in monocytic THP-1 cells. Cumulative total ROS production of 2 × 10^5^ monocytic THP-1 cells in response to ops. *S. aureus* (at least 5 bacteria per cell; *n* = 4) and ops. zymosan (150 µg/ml; *n* = 3) following 30 min pretreatment with the indicated doses of BIO-11006 (mean ± SD). (**C**) BIO-11006 inhibits PMA-induced ROS production in monocytic and neutrophilic cells. 100 nM PMA-induced cumulative total ROS production of monocytic THP-1 and PLB-985 cells (3 × 10^5^), neutrophilic PLB-985 cells, and primary human monocytes (2 × 10^5^) pretreated with BIO-11006 (mean ± SD, *n* = 3 each). (**D**) BIO-11006 inhibits PMA-induced ROS production in monocytic THP-1- and PLB-985-derived MARCKS WT and KO cells. PMA- and ops. zymosan-induced cumulative total ROS production of monocytic THP-1- and PLB-985-derived MARCKS WT and KO cells pretreated with 1 mM BIO-11006 (mean ± SD, *n* = 3; for PLB-985-derived WT and KO cells, each data point represents the mean of WT1 and WT2 or KO1 and KO2, respectively). Stimulus-induced cumulative total ROS levels in untreated controls (i.e., without BIO-11006) were set as 1 (dotted line). *p ≤ 0.05; **p ≤ 0.01 in comparison to the untreated control; unpaired t test with Welch’s correction

As an alternative to MANS, its shorter derivative BIO-11006 was applied (Fig. 6A). First, the efficacy of BIO-11006 on calcitriol-differentiated THP-1 cells was assessed in dose-response experiments (10 µM −1 mM). Interestingly, BIO-11006 did not affect total ROS production in ops. *S. aureus*- and ops. zymosan-stimulated samples (Fig. 6B). In PMA-activated THP-1, however, a clear dose-dependent effect on ROS production could be observed, with a significant reduction at 1 mM BIO-11,006 (Fig. 6C), suggesting that BIO-11006 has differential effects when interfering with different ROS-inducing stimuli. Further dose-response experiments demonstrated that 1 mM BIO-11006 significantly decreased PMA-provoked ROS generation in PLB-985 monocytic cells, PLB-985 neutrophils, as well as primary human monocytes (Fig. 6C). In the latter cases, significantly reduced ROS levels were also observed in samples treated with 500 µM BIO-11006. To test whether BIO-11006 (like MANS) affects both MARCKS WT and KO cells, THP-1- and PLB-985-derived monocytic WT and KO cells were treated with 1 mM BIO-11006, before ROS production was induced by PMA or ops. zymosan. Again, no reduction of ROS levels could be observed in zymosan-stimulated THP-1- or PLB-985-derived WT and KO cells, while PMA-stimulated WT and KO cells exhibited reduced ROS in the presence of BIO-11006 (Fig. 6D). As in the case of MANS, the influence of BIO-11006 on ROS levels was comparable in WT and KO cells and showed no differential effects.

### Monocytic chemotaxis is inhibited by MANS in both MARCKS WT and KO cells

**Fig. 7:**
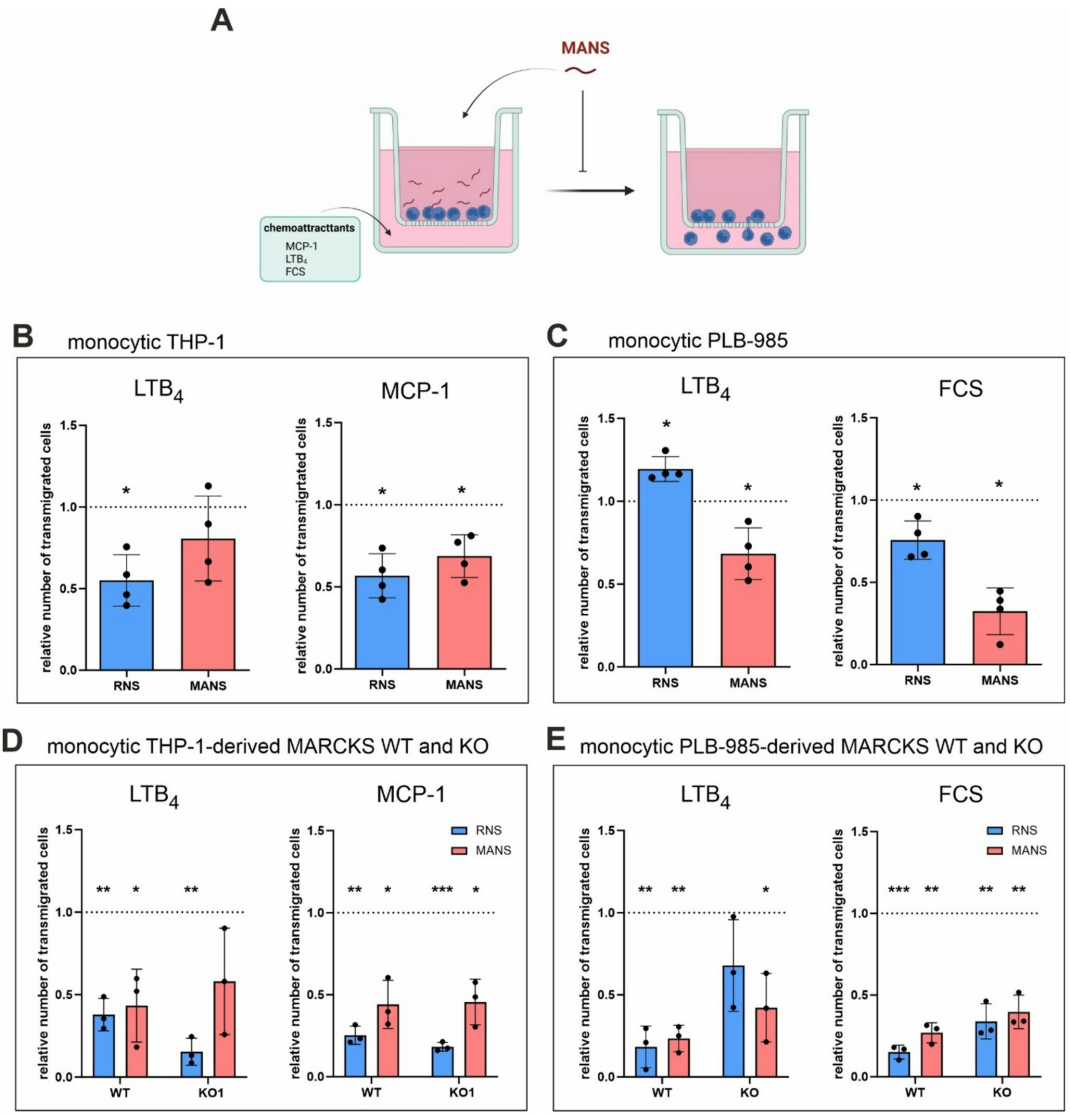
MANS inhibits monocytic transmigration in MARCKS WT and KO cells. (**A**) Experimental design. Differentiated THP-1 (2 × 10^5^ cells), PLB-985 (3 × 10^5^ cells), and THP-1-/PLB-985-derived MARCKS WT and KO cells were preincubated with 100 µM MANS or RNS. Transmigration in response to 100 ng/ml LTB_4_, 50 ng/ml MCP-1, and/or 10% FCS was measured in transwell assays. Created with BioRender.com. (**B**, **C**) MANS and RNS inhibit chemoattractant-induced transmigration of monocytic THP-1 and PLB-985 cells. Relative number of transmigrated monocytic THP-1 (**B**) and PLB-985 (**C**) in response to the indicated chemoattractants following pretreatment with MANS or RNS (mean ± SD, *n* = 4). (**D**, **E**) MANS and RNS inhibit chemoattractant-induced transmigration of monocytic THP-1- and PLB-985-derived MARCKS WT and KO cells. Relative number of transmigrated monocytic THP-1- (**D**) and PLB-985-derived (**E**) WT and KO cells in response to the indicated chemoattractants following pretreatment with MANS or RNS (mean ± SD, *n* = 3; for PLB-985-derived WT and KO cells, each data point represents the mean of WT1 and WT2 or KO1 and KO2, respectively). Chemoattractant-induced transmigration in solvent-treated controls was set as 1 (dotted line). *p ≤ 0.05; **p ≤ 0.01 in comparison to the solvent-treated control; unpaired t test with Welch’s correction

To assess whether additional cellular processes of monocytes are inhibited by MANS in an unspecific manner, migration was selected as an additional MANS-sensitive read-out parameter [22, 31]. First, MANS-modulated chemotaxis of THP-1 and PLB-985 monocytic cells was measured in response to leukotriene B_4_ (LTB_4_) as well as monocyte chemoattractant protein 1 (MCP-1) or FCS (in the case of PLB-985, which – as a subclone of HL-60 cells [39] – do virtually not express the MCP-1 receptor CCR2 [40]) (Fig. 7A). The application of MANS reduced (in most cases: significantly) chemoattractant-dependent migration of THP-1 (Fig. 7B) and PLB-985 monocytes (Fig. 7C). Interestingly, the control peptide RNS also inhibited monocytic chemotaxis (Fig. 7B, C; with the exception of LTB_4_-induced PLB-985 transmigration, Fig. 7C) suggesting an unspecific inhibitory effect of RNS on migration, at least under certain conditions. Afterwards, the impact of MANS and RNS on THP-1- and PLB-985-derived MARCKS WT and KO cells was addressed. In THP-1-derived WT and KO monocytes, MANS and RNS suppressed LTB_4_- and MCP-1-triggered chemotaxis, while no differences between MANS-treated WT and KO cells could be observed (Fig. 7D). PLB-985-derived WT and KO cells exhibited an equivalent reduction of transmigration when treated with MANS and RNS (Fig. 7E). Though the remarkably clear suppressive effect of RNS on monocytic chemotaxis is surprising, MANS demonstrated again that it provokes the same effects in both WT and KO cells. Thus, this set of experiments illustrates that MARCKS-independent properties of MANS do not only affect ROS production, but further monocytic cell functions as shown for chemotaxis/migration.

## Discussion

Since its identification and characterization as a major PKC substrate [41] and actin-binding protein [3], MARCKS proved to be involved in various cellular processes including intracellular signaling and transportation, proliferation, motility and migration, adhesion, spreading, and secretion. Thus, MARCKS participates in molecular mechanisms underlying a variety of physiological processes such as growth and development, neurological functions, regeneration, and inflammation [1, 2]. In consequence, alterations of MARCKS’ level, function, and localization may contribute to the development and progression of multiple diseases. For instance, MARCKS has been described to play a role in autoimmunity, neurological disorders [13], solid tumors [1] and hematological malignancies [7], fibrosis [15], as well as respiratory diseases [20]. Recently, MARCKS has been proposed as a potential biomarker for ischemic stroke [42] and a prognostic marker for endometrial cancer [43]. Its involvement in this plethora of mechanisms, processes, and functions renders MARCKS a promising target for pharmacological intervention. However, due to the absence of enzymatic activity, the development of small molecule inhibitors is not possible. A breakthrough was achieved when it became evident that peptides derived from the MARCKS sequence are able to reliably inhibit MARCKS-associated functions, as demonstrated for the ED peptide [17] and MANS [21]. Therefore, these peptides are generally regarded as MARCKS-specific inhibitors [20].

Though MANS turned out to be a powerful tool in characterizing MARCKS and its functions [25], it was unsuitable for pharmaceutical use due to insufficient solubility and stability [20], an insight that led to the development of the highly soluble BIO-11006 [26]. This derivative of MANS is shorter and not myristoylated, which enhances its stability and solubility [20] and facilitates its applicability [28]. However, beneficial effects on inflammatory, respiratory, and/or malignant diseases have been shown for both MANS [14, 23, 24] and BIO-11,006 [26–28], esp. in the mouse model. Neither BIO-11006 [20] nor MANS or its control peptide RNS appear to exhibit cytotoxic activity [21, 44] or to impair metabolic processes [20, 21]. In addition, no indication for mutagenicity or clastogenicity could be observed [20]. Accordingly, BIO-11006 is the subject of clinical phase II studies for lung diseases [19, 29].

As we have shown before in monocytic cells that MARCKS deficiency significantly impairs PMA- and ops. gram-negative bacteria (i.e., *E. coli*-)induced ROS production [10], the present study was originally designed to test whether ROS production by alternative stimuli (i.e., ops. gram-positive *S. aureus* and ops. zymosan) also involves MARCKS and whether MARCKS inhibition *via* MANS and BIO-11006 is sufficient to effectively suppress monocytic total ROS generation. With regard to antimicrobial defense and immune cell activation, intracellular ROS (which are mainly produced by mitochondria and phagosomes) are more relevant than total ROS [45]. In our previous study [10], however, we have shown that MARCKS deficiency affects total and intracellular ROS generation similarly, which strongly suggests that under our conditions, changes in total ROS levels adequately reflect alterations in intracellular ROS as well. Moreover, intracellular ROS amounts are considerably lower than total ROS levels and stand out less clearly against the background in monocytic cells. Therefore, we differentiated premonocytic THP-1, myeloid PLB-985, and THP-1- and PLB-985-derived MARCKS WT and KO cells with calcitriol towards the monocytic phenotype [37] and addressed total ROS production as major read-out in these cells. As expected, our initial experiments demonstrated that ROS formation in MARCKS KO cells is significantly inhibited irrespective of the stimulus used. Further, MANS - but not the control peptide - reduced (in most cases: significantly) ROS levels in PMA-, ops. *E. coli*-, ops. *S. aureus-*, and ops. zymosan-challenged monocytic THP-1 and PLB-985 cells as well as primary human monocytes thus reflecting the effect earlier observed in KO cells [10]. Long-term TNF preincubation [36] remarkably enhanced stimulation-induced total ROS production in monocytic THP-1 cells, which is consistent with our previous study [10], but had no influence on MANS-dependent inhibition of ROS formation. DMSO-differentiated PLB-985 neutrophils, which were tested in a set of experiments, were also prone to an equivalent MANS-dependent inhibition of ROS, which is in good agreement with reports describing that MANS significantly inhibited the immune complex-mediated oxidative burst of equine [46] and human neutrophils [47]. In bovine neutrophils, however, *S. typhimurium*-dependent ROS production was increased in the presence of low concentrations of MANS (up to 25 µM), while 100 µM (as used in our present study) had no significant impact [48], suggesting that species- and/or stimulus-specific effects may play a role in this context. A major influence of the ROS-inducing stimulus on MANS-dependent modulation of ROS formation would fit well to our observation that in most cases, MANS had a more pronounced effect on ops. *E. coli*-, *S. aureus*-, and zymosan-induced ROS levels, while BIO-11006 predominantly affected PMA-activated ROS production. Together with the literature [46–48], this might suggest that MANS mediates its molecular function in dependency of the respective intracellular pathway activated.

Though the specific mechanism(s) of action remain(s) unclear, it was proposed that MANS might exert its function by competing with MARCKS for membrane binding [21], thus displacing MARCKS from the membrane [49], or by directly interacting with the MARCKS protein [44]. Moreover, both MANS and BIO-11006 are supposed to inhibit MARCKS phosphorylation [19]. However, irrespective of the actual mechanism, the concept of using MARCKS-derived peptides as MARCKS inhibitors implies that these peptides should not exert an inhibitory effect in the absence of their specific target, e.g., in MARCKS KO cells. Therefore, it was surprising to detect significantly decreased ROS levels in MANS-treated THP-1- and PLB-985-derived MARCKS KO cells, i.e., cells that were intended to serve as negative controls. This observation suggests that MANS (also) possesses MARCKS-independent activities, at least in myelomonocytic cells. To date, it is unclear whether these MARCKS-unspecific effects represent an additional mechanistic feature of MANS (either permanently active or exclusively emerging in the absence of MARCKS) - or whether MANS has no or only limited MARCKS-specific activity and mediates its physiological effects *via* not yet identified target(s) presumably downstream of MARCKS.

To further characterise the unexpected activity of MANS in MARCKS KO cells, the modulatory influence of TNF was assessed. Consistent with the priming effect in neutrophils and polymorphonuclear leukocytes [50], TNF significantly enhanced ROS production by monocytic THP-1 as well as THP-1-derived WT and KO cells in response to a selection of stimuli, i.e., ops. *E. coli* and ops. zymosan. In KO cells, however, even TNF-elevated ROS amounts remained on a lower level than in the WT, thus reflecting our previous investigations [10]. Again, MANS treatment inhibited (TNF-elevated) ROS generation consistently in WT and KO cells. This indicates that in MARCKS KO cells, TNF mediates priming, but neither a compensatory effect on reduced ROS levels nor a differential effect on MANS-dependent ROS inhibition.

Since BIO-11006 consists of the active region of MANS [27], it represents a suitable alternative to that peptide. Using BIO-11006, we observed a (dose-dependent) inhibitory effect similar to MANS when PMA was applied for inducing ROS production in monocytic THP-1 cells. Equivalent results were obtained from monocytic and neutrophilic PLB-985 cells as well as primary human monocytes. In contrast, no suppression could be detected, when ROS were induced by ops. *S. aureus* or zymosan. This outcome was unexpected, as MANS and BIO-11006 generally show similar effects, e.g., on mucus secretion, inflammation [20], and cancer progression [19]. Discrepancies between both substances are usually described in terms of their potency, with BIO-11006 outperforming MANS due to its superior applicability [19, 27]. However, the considerable differences between MANS and BIO-11006 in cells treated with physiological stimuli suggest that the peptides affect distinct intracellular pathways *via* differential molecular mechanisms. One could speculate that length and the presence of the myristoyl group may contribute to differing mechanism of action [20]. Nonetheless, like MANS, BIO-11006 results in reduced ROS levels when applied to (PMA-stimulated) THP-1- and PLB-985-derived MARCKS KO cells, indicating that it is also able to act in a MARCKS-independent manner.

To clarify whether the target-independent properties of MANS are specific for ROS generation or of general importance for monocytes, cell migration was analyzed. In the literature, migration is characterised as another MARCKS-associated cellular function prone to inhibition by MANS [22, 23, 28, 31]. In the present study, a similar inhibition of chemoattractant-induced migration by MANS was observed for monocytic THP-1 and PLB-985 cells as well as MARCKS WT and KO cells (with the exception of LTB_4_-attracted THP-1 and THP-1-derived KO cells, which showed a clear, but not statistically significant tendency towards reduced chemotaxis). Interestingly, under most conditions, the scrambled control RNS had comparable negative effects on monocytic chemotaxis, though RNS did not show a significant influence on a variety of parameters in other studies including murine mucin secretion [14, 21], neutrophil infiltration, and cytokine production [26] as well as neutrophil [49], macrophage [31], and lung cancer cell migration [23]. This suggests an unspecific inhibitory effect of RNS on chemotaxis, at least of human monocytic cells, under certain conditions. However, irrespective of the astonishing consequences of RNS treatment, MANS’ remarkable inhibition of migration of all monocytic cell types analysed, including MARCKS KO cells, promotes our assumption that MARCKS-derived peptides possess MARCKS-independent functions. Thus, the ability to affect MARCKS-associated cellular processes in monocytes in a MARCKS-independent manner appears to be a general feature of MANS. It is therefore conceivable that the beneficial effects of MANS and BIO-11006 observed under several pathophysiologic conditions [14, 23, 24, 26–28] can even be achieved in the absence of MARCKS.

To date, MARCKS-independent effects of MANS have only been described in a single study reporting that MANS inhibited hexosaminidase secretion from both MARCKS WT and KO murine embryonic hepatic-derived mast cells [51]. Together with our own observations, these data challenge to some extent the assumption that MARCKS-derived inhibitor peptides are exclusively directed towards MARCKS. Rather, our findings argue for a concept in which these peptides are able to inhibit MARCKS-associated monocytic processes *via* MARCKS-independent - and presumably differential - mechanism(s), either in addition to or independent of MARCKS-specific effects.

## Conclusion

Here we show that MARCKS inhibitor peptides MANS and (to a lesser extent) BIO-11006 are able to impair MARCKS-associated monocytic cell functions such as ROS production or migration, thus mimicking the effects of MARCKS deficiency. Equivalent effects observed in MARCKS KO cells, however, suggest that these peptides also possess MARCKS-independent activities and may affect MARCKS-associated cellular processes by MARCKS-independent mechanisms. While the basic effectiveness of MANS and BIO-11006 is not contested by our results, an extension of the inhibitor peptides’ mode of action - at least in monocytic cells - has to be taken into consideration.

## Data Availability

Data will be made available on request.

## References

[1] Fong LWR, Yang DC, Chen CH (2017) Myristoylated alanine-rich C kinase substrate (MARCKS): a multirole signaling protein in cancers. Cancer Metastasis Rev 36:737–747. 10.1007/s10555-017-9709-6 29039083 10.1007/s10555-017-9709-6

[2] El Amri M, Fitzgerald U, Schlosser G (2018) MARCKS and MARCKS-like proteins in development and regeneration. J Biomed Sci 25:43. 10.1186/s12929-018-0445-1 29788979 10.1186/s12929-018-0445-1 PMC5964646

[3] Hartwig JH, Thelen M, Rosen A, Janmey PA, Nairn AC, Aderem A (1992) MARCKS is an actin filament crosslinking protein regulated by protein kinase C and calcium-calmodulin. Nature 356:618–622. 10.1038/356618a0 1560845 10.1038/356618a0

[4] Glaser M, Wanaski S, Buser CA, Boguslavsky V, Rashidzada W, Morris A, Rebecchi M, Scarlata SF, Runnels LW, Prestwich GD, Chen J, Aderem A, Ahn J, McLaughlin S (1996) Myristoylated alanine-rich C kinase substrate (MARCKS) produces reversible inhibition of phospholipase C by sequestering phosphatidylinositol 4,5-bisphosphate in lateral domains. J Biol Chem 271:26187–26193. 10.1074/jbc.271.42.26187 8824266 10.1074/jbc.271.42.26187

[5] Xu XH, Deng CY, Liu Y, He M, Peng J, Wang T, Yuan L, Zheng ZS, Blackshear PJ, Luo ZG (2014) MARCKS regulates membrane targeting of Rab10 vesicles to promote axon development. Cell Res 24:576–594. 10.1038/cr.2014.33 24662485 10.1038/cr.2014.33 PMC4011341

[6] Ziemba BP, Burke JE, Masson G, Williams RL, Falke JJ (2016) Regulation of PI3K by PKC and MARCKS: single-molecule analysis of a reconstituted signaling pathway. Biophys J 110:1811–1825. 10.1016/j.bpj.2016.03.001 27119641 10.1016/j.bpj.2016.03.001 PMC4850241

[7] Iyer DN, Faruq O, Zhang L, Rastgoo N, Liu A, Chang H (2021) Pathophysiological roles of myristoylated alanine-rich C-kinase substrate (MARCKS) in hematological malignancies. Biomark Res 9:34. 10.1186/s40364-021-00286-9 33958003 10.1186/s40364-021-00286-9 PMC8101130

[8] Lee SM, Suk K, Lee WH (2015) Myristoylated alanine-rich C kinase substrate (MARCKS) regulates the expression of proinflammatory cytokines in macrophages through activation of p38/JNK MAPK and NF-kappaB. Cell Immunol 296:115–121. 10.1016/j.cellimm.2015.04.004 25929183 10.1016/j.cellimm.2015.04.004

[9] Issara-Amphorn J, Sjoelund VH, Smelkinson M, Montalvo S, Yoon SH, Manes NP, Nita-Lazar A (2023) Myristoylated, alanine-rich C-kinase substrate (MARCKS) regulates toll-like receptor 4 signaling in macrophages. Sci Rep 13:19562. 10.1038/s41598-023-46266-x 37949888 10.1038/s41598-023-46266-x PMC10638260

[10] Huber R, Diekmann M, Hoffmeister L, Kuhl F, Welz B, Brand K (2022) MARCKS is an essential regulator of reactive oxygen species production in the monocytic cell type. Antioxidants 11:1600. 10.3390/antiox11081600 36009319 10.3390/antiox11081600 PMC9404745

[11] Nakanishi A, Wada Y, Kitagishi Y, Matsuda S (2014) Link between PI3K/AKT/PTEN pathway and NOX proteinin diseases. Aging Dis 5:203–211. 10.14336/AD.2014.0500203 24900943 10.14336/AD.2014.0500203 PMC4037312

[12] Welz B, Bikker R, Junemann J, Christmann M, Neumann K, Weber M, Hoffmeister L, Preuss K, Pich A, Huber R, Brand K (2019) Proteome and phosphoproteome analysis in TNF long term-exposed primary human monocytes. Int J Mol Sci 20:e1241. 10.3390/ijms20051241 10.3390/ijms20051241 PMC6429050 30871024

[13] Chen Z, Zhang W, Selmi C, Ridgway WM, Leung PSC, Zhang F, Gershwin ME (2021) The myristoylated alanine-rich C-kinase substrates (MARCKS): a membrane-anchored mediator of the cell function. Autoimmun Rev 20:102942. 10.1016/j.autrev.2021.102942 34509657 10.1016/j.autrev.2021.102942 PMC9746065

[14] Singer M, Martin LD, Vargaftig BB, Park J, Gruber AD, Li Y, Adler KB (2004) A MARcks-related peptide blocks mucus hypersecretion in a mouse model of asthma. Nat Med 10:193–196. 10.1038/nm983 14716307 10.1038/nm983

[15] Yang DC, Li JM, Xu J, Oldham J, Phan SH, Last JA, Wu R, Chen CH (2019) Tackling MARCKs-PIP3 circuit attenuates fibroblast activation and fibrosis progression. FASEB J 33:14354–14369. 10.1096/fj.201901705R 31661644 10.1096/fj.201901705R PMC6894092

[16] Yadav V, Jena MK, Parashar G, Parashar NC, Joshi H, Ramniwas S, Tuli HS (2024) Emerging role of MicroRNAs as regulators of protein kinase C substrate MARCKS and MARCKSL1 in cancer. Exp Cell Res 434:113891. 10.1016/j.yexcr.2023.113891 38104645 10.1016/j.yexcr.2023.113891

[17] Graff JM, Young TN, Johnson JD, Blackshear PJ (1989) Phosphorylation-regulated calmodulin binding to a prominent cellular substrate for protein kinase C. J Biol Chem 264:21818–21823. 10.1016/S0021-9258(20)88257-X 2557340

[18] Kim J, Blackshear PJ, Johnson JD, McLaughlin S (1994) Phosphorylation reverses the membrane association of peptides that correspond to the basic domains of MARCKS and neuromodulin. Biophys J 67:227–237. 10.1016/S0006-3495(94)80473-4 7918991 10.1016/S0006-3495(94)80473-4 PMC1225353

[19] Yadav V, Sharma AK, Parashar G, Parashar NC, Ramniwas S, Jena MK, Tuli HS, Yadav K (2023) Patent landscape highlighting therapeutic implications of peptides targeting myristoylated alanine-rich protein kinase-C substrate (MARCKS). Expert Opin Ther Pat 33:445–454. 10.1080/13543776.2023.2240020 37526024 10.1080/13543776.2023.2240020

[20] Sheats MK, Yin Q, Fang S, Park J, Crews AL, Parikh I, Dickson B, Adler KB (2019) MARCKS and lung disease. Am J Respir Cell Mol Biol 60:16–27. 10.1165/rcmb.2018-0285TR 30339463 10.1165/rcmb.2018-0285TR PMC6348718

[21] Li Y, Martin LD, Spizz G, Adler KB (2001) MARCKS protein is a key molecule regulating mucin secretion by human airway epithelial cells in vitro. J Biol Chem 276:40982–40990. 10.1074/jbc.M105614200 11533058 10.1074/jbc.M105614200

[22] Ott LE, Sung EJ, Melvin AT, Sheats MK, Haugh JM, Adler KB, Jones SL (2013) Fibroblast migration is regulated by myristoylated alanine-rich C-kinase substrate (MARCKS) protein. PLoS ONE 8:e66512. 10.1371/journal.pone.0066512 23840497 10.1371/journal.pone.0066512 PMC3686679

[23] Chen CH, Thai P, Yoneda K, Adler KB, Yang PC, Wu R (2014) A peptide that inhibits function of myristoylated Alanine-Rich C kinase substrate (MARCKS) reduces lung cancer metastasis. Oncogene 33:3696–3706. 10.1038/onc.2013.336 23955080 10.1038/onc.2013.336 PMC4631387

[24] Takashi S, Park J, Fang S, Koyama S, Parikh I, Adler KB (2006) A peptide against the N-terminus of myristoylated alanine-rich C kinase substrate inhibits degranulation of human leukocytes *in vitro*. Am J Respir Cell Mol Biol 34:647–652. 10.1165/rcmb.2006-0030RC 16543603 10.1165/rcmb.2006-0030RC PMC2644225

[25] Ferdous J, Naitou K, Shiraishi M (2024) A peptide against the N-terminus of myristoylated alanine-rich C kinase substrate promotes neuronal differentiation in SH-SY5Y human neuroblastoma cells. J Vet Med Sci 86:1136–1144. 10.1292/jvms.24-0276 39343539 10.1292/jvms.24-0276 PMC11569876

[26] Damera G, Jester WF, Jiang M, Zhao H, Fogle HW, Mittelman M, Haczku A, Murphy E, Parikh I, Panettieri RA Jr (2010) Inhibition of myristoylated alanine-rich C kinase substrate (MARCKS) protein inhibits ozone-induced airway neutrophilia and inflammation. Exp Lung Res 36:75–84. 10.3109/01902140903131200 10.3109/01902140903131200 PMC4064305 20205598

[27] Yin Q, Fang S, Park J, Crews AL, Parikh I, Adler KB (2016) An inhaled inhibitor of myristoylated Alanine-Rich C kinase substrate reverses LPS-induced acute lung injury in mice. Am J Respir Cell Mol Biol 55:617–622. 10.1165/rcmb.2016-0236RC 27556883 10.1165/rcmb.2016-0236RC PMC5105187

[28] Agrawal A, Murphy ECI, Park J, Adler KB, Parikh I (2011) Aerosolized BIO-11006, a novel MARCKS- related peptide, improves airway obstruction in a mouse model of mucus hypersecretion. J Epithel Biol Pharmacol 4:1–6. 10.2174/1875044301104010001

[29] U.S. National Library of Medicine Database of Clinical Trails (2025) [https://clinicaltrials.gov/]. (Accessed on 20 June

[30] Chiu CL, Zhao H, Chen CH, Wu R, Brooks JD (2022) The role of MARCKS in metastasis and treatment resistance of solid tumors. Cancers (Basel). 10.3390/cancers14194925 36230850 10.3390/cancers14194925 PMC9563266

[31] Green TD, Park J, Yin Q, Fang S, Crews AL, Jones SL, Adler KB (2012) Directed migration of mouse macrophages in vitro involves myristoylated alanine-rich C-kinase substrate (MARCKS) protein. J Leukoc Biol 92:633–639. 10.1189/jlb.1211604 22623357 10.1189/jlb.1211604 PMC3427602

[32] Ran FA, Hsu PD, Wright J, Agarwala V, Scott DA, Zhang F (2013) Genome engineering using the CRISPR-Cas9 system. Nat Protoc 8:2281–2308. 10.1038/nprot.2013.143 24157548 10.1038/nprot.2013.143 PMC3969860

[33] Doench JG, Fusi N, Sullender M, Hegde M, Vaimberg EW, Donovan KF, Smith I, Tothova Z, Wilen C, Orchard R, Virgin HW, Listgarten J, Root DE (2016) Optimized SgRNA design to maximize activity and minimize off-target effects of CRISPR-Cas9. Nat Biotechnol 34:184–191. 10.1038/nbt.3437 26780180 10.1038/nbt.3437 PMC4744125

[34] Welz B, Bikker R, Hoffmeister L, Diekmann M, Christmann M, Brand K, Huber R (2021) Activation of GSK3 prevents termination of TNF-induced signaling. J Inflamm Res 14:1717–1730. 10.2147/JIR.S300806 33986607 10.2147/JIR.S300806 PMC8111165

[35] Gunther J, Vogt N, Hampel K, Bikker R, Page S, Muller B, Kandemir J, Kracht M, Dittrich-Breiholz O, Huber R, Brand K (2014) Identification of two forms of TNF tolerance in human monocytes: differential Inhibition of NF-kappaB/AP-1- and PP1-associated signaling. J Immunol 192:3143–3155. 10.4049/jimmunol.1301610 24574500 10.4049/jimmunol.1301610

[36] Bikker R, Christmann M, Preuss K, Welz B, Friesenhagen J, Dittrich-Breiholz O, Huber R, Brand K (2017) TNF phase III signalling in tolerant cells is tightly controlled by A20 and CYLD. Cell Signal 37:123–135. 10.1016/j.cellsig.2017.06.009 28629782 10.1016/j.cellsig.2017.06.009

[37] Huber R, Panterodt T, Welz B, Christmann M, Friesenhagen J, Westphal A, Pietsch D, Brand K (2015) C/EBPbeta-LAP*/LAP expression is mediated by RSK/eIF4B-Dependent signalling and boosted by increased protein stability in models of monocytic differentiation. PLoS ONE 10:e0144338. 10.1371/journal.pone.0144338 26646662 10.1371/journal.pone.0144338 PMC4672875

[38] Tucker KA, Lilly MB, Heck L Jr., Rado TA (1987) Characterization of a new human diploid myeloid leukemia cell line (PLB-985) with granulocytic and monocytic differentiating capacity. Blood 70:372–378. 10.1182/blood.V70.2.372.372 3475136

[39] Drexler HG, Dirks WG, Matsuo Y, MacLeod RA (2003) False leukemia-lymphoma cell lines: an update on over 500 cell lines. Leukemia 17:416–426. 10.1038/sj.leu.2402799 12592342 10.1038/sj.leu.2402799

[40] The Human Protein Atlas (2025) [https://www.proteinatlas.org]. (Accessed on 20 June

[41] Stumpo DJ, Graff JM, Albert KA, Greengard P, Blackshear PJ (1989) Molecular cloning, characterization, and expression of a cDNA encoding the 80- to 87-kDa myristoylated alanine-rich C kinase substrate: a major cellular substrate for protein kinase C. Proc Natl Acad Sci U S A 86:4012–4016. 10.1073/pnas.86.11.4012 2726763 10.1073/pnas.86.11.4012 PMC287378

[42] Xu L, Wang P, Yang L, Liu Y, Li X, Yin Y, Lan C (2025) Neurotrophic factor biomarkers for ischemic stroke diagnosis and mechanistic insights. Sci Rep 15:11906. 10.1038/s41598-025-86935-7 40195336 10.1038/s41598-025-86935-7 PMC11977241

[43] Wan C, Zhang L, Yu T, Lu H, Xiao H, Du J (2025) Identification of key genes underlying radiosensitivity and radioresistance in endometrial cancer through integrated bioinformatics analysis. Front Genet 16:1469610. 10.3389/fgene.2025.1469610 39926275 10.3389/fgene.2025.1469610 PMC11802559

[44] Machlus KR, Wu SK, Stumpo DJ, Soussou TS, Paul DS, Campbell RA, Kalwa H, Michel T, Bergmeier W, Weyrich AS, Blackshear PJ, Hartwig JH, Italiano JE Jr (2016) Synthesis and dephosphorylation of MARCKS in the late stages of megakaryocyte maturation drive proplatelet formation. Blood 127:1468–1480. 10.1182/blood-2015-08-663146 26744461 10.1182/blood-2015-08-663146 PMC4797023

[45] Shekhova E (2020) Mitochondrial reactive oxygen species as major effectors of antimicrobial immunity. PLoS Pathog 16:e1008470. 10.1371/journal.ppat.1008470 32463825 10.1371/journal.ppat.1008470 PMC7255592

[46] Sheats MK, Pescosolido KC, Hefner EM, Sung EJ, Adler KB, Jones SL (2014) Myristoylated alanine rich C kinase substrate (MARCKS) is essential to beta2-integrin dependent responses of equine neutrophils. Vet Immunol Immunopathol 160:167–176. 10.1016/j.vetimm.2014.04.009 24857637 10.1016/j.vetimm.2014.04.009 PMC4108539

[47] Conley H, Till RL, Berglund AK, Jones SL, Sheats MK (2023) A myristoylated alanine-rich C-kinase substrate (MARCKS) inhibitor peptide attenuates neutrophil outside-in beta(2)-integrin activation and signaling. Cell Adh Migr 17:1–16. 10.1080/19336918.2023.2233204 37439125 10.1080/19336918.2023.2233204 PMC10348033

[48] Conley HE, Brown CF, Westerman TL, Elfenbein JR, Sheats MK (2024) MARCKS inhibition alters bovine neutrophil responses to *Salmonella typhimurium*. Biomedicines 12:442. 10.3390/biomedicines12020442 38398044 10.3390/biomedicines12020442 PMC10886653

[49] Eckert RE, Neuder LE, Park J, Adler KB, Jones SL (2010) Myristoylated alanine-rich C-kinase substrate (MARCKS) protein regulation of human neutrophil migration. Am J Respir Cell Mol Biol 42:586–594. 10.1165/rcmb.2008-0394OC 19574534 10.1165/rcmb.2008-0394OC PMC2874444

[50] Volk AP, Barber BM, Goss KL, Ruff JG, Heise CK, Hook JS, Moreland JG (2011) Priming of neutrophils and differentiated PLB-985 cells by pathophysiological concentrations of TNF-alpha is partially oxygen dependent. J Innate Immun 3:298–314. 10.1159/000321439 21088376 10.1159/000321439 PMC3128147

[51] Haddock BJ, Zhu Y, Doyle SP, Abdullah LH, Davis CW (2014) Role of MARCKS in regulated secretion from mast cells and airway goblet cells. Am J Physiol Lung Cell Mol Physiol 306:L925–936. 10.1152/ajplung.00213.2013 24705720 10.1152/ajplung.00213.2013

